# The phytochemical properties, pharmacological effects and traditional uses of *Actinidia eriantha* Benth.: A review

**DOI:** 10.3389/fphar.2022.959900

**Published:** 2022-08-19

**Authors:** Shiyu Wang, Xiaoqi Gao, Qingmei Sun, Yichun Zhu, Luping Qin, Bo Zhu

**Affiliations:** School of Pharmaceutical Sciences, Zhejiang Chinese Medical University, Hangzhou, China

**Keywords:** *Actinidia eriantha*, traditional use, phytochemistry, pharmacology, quality control

## Abstract

*Actinidia eriantha* Benth. (Called *Maohuamihoutao* in China) is a plant that has been utilized as a heat-clearing drug in *She* ethnic minority group for a long time in China. Specifically, it has been involved in the treatment of stomach cancer, colon cancer, cirrhosis with ascites, chronic hepatitis, leukemia, rectal prolapse, hernia and uterine prolapse. Pharmacological research provides partial evidence for the traditional use of *A. eriantha* and might have demonstrated the folk utilization of *A. eriantha* to combat many cancers. Crude extracts and relatively pure components of *A. eriantha* possess a variety of pharmacological activities, including anti-cancer, immunoregulatory, anti-angiogenic, neuroprotective, anti-inflammatory, and antioxidant activities. In addition, over 104 chemical substances have been determined from *A. eriantha*, involving terpenoids, alcohols, phenolics, aldehydes, organic acids, flavonoids glycosides, ketones, and glucoside. The existing literature reveals that a large proportion of the therapeutic effects of *A. eriantha* were rendered by the polysaccharides. However, the mechanisms of action and the structure-function correlations of these compounds, as well as the synergistic and antagonistic effects between them, need to be investigated further. Therefore, we propose that future studies on *A. eriantha* should focus on comprehensively assessing its medicinal quality, exploring its multi-target nature using network pharmacology approaches, and evaluating its long-term toxicity and efficacy *in vivo*.

## Introduction

The genus *Actinidia* (Actinidiaceae) includes more than fifty-two perennial herbal species broadly distributed all over China (Flora of China Editorial Committee, 2015). Among them, *Actinidia eriantha* Benth. (AE) is a liana species commonly found in regions in the temperate zone (Xu et al., 2009a). The roots of AE (Actinidiaceae) have been utilized to treat various cancers, such as gastric cancer, mastocarcinoma, esophagus cancer, cervical carcinoma, nasopharyngeal cancer, and prostatic cancer, as well as cirrhosis with ascites, chronic hepatitis etc. in traditional Chinese medicine (Yu et al., 2017; Chen et al., 2019a). The AE roots are shown to have many biological activities, including anti-tumor (Xu et al., 2009b), immunoregulatory (Xu et al., 2009b), anti-angiogenic (Wu et al., 2017), neuroprotective (Cho et al., 2021), anti-inflammatory (Kim et al., 2018) and antioxidant (Huang et al., 2020) effects. Phytochemical investigations exhibited that AE contains a rich phytochemical like terpenoids, alcohols, phenolics, aldehydes, organic acids, flavonoids glycosides, ketones, glucoside. However, only a few of these compounds have been subjected to bioactivity studies, and their corresponding structures have not been adequately summarized and comprehensively presented in other publications. Although AE has showed its efficacy against cancers, its extracts contain chemical substances with so far undefined toxicity and biosafety. Moreover, both quality control research and rigorous pharmacological assessments of the associations between the extracts and the traditional utilization of AE are lacking.

We herein consistently organize the unsorted information on botanical characteristics, traditional uses, phytochemical properties, pharmacological activities, probable molecular mechanisms, quality control and biosafety of AE. Our paper displays phytochemical and pharmacological research on AE to testify its traditional application in the treatment of diseases. The information summarized in this paper will guide the design of future *in vivo* and clinical studies on AE and the development of new medicine containing AE or its active components.

## Materials and methods

Research available on AE was retrieved from electronic databases of Baidu Scholar, Google Scholar, SciFinder, PubMed, ScienceDirect, Web of Science, and Springer using the keywords of “*Actinidia eriantha,*” “biological activity,” “phytochemistry,” “pharmacology,” “secondary metabolites,” “medicinal uses,” “safety,” “quality control,” “ethnobotanical survey,” “toxicology,” and related terms. Papers published on scientific journals, Chinese herbal medicine books and magazines, as well as thesises, were obtained. We utilized The Plant List (www.theplantlist.org) database to verify the nomenclature and acquire information on AE subspecies.

## Botanical characteristics

**FIGURE 1:**
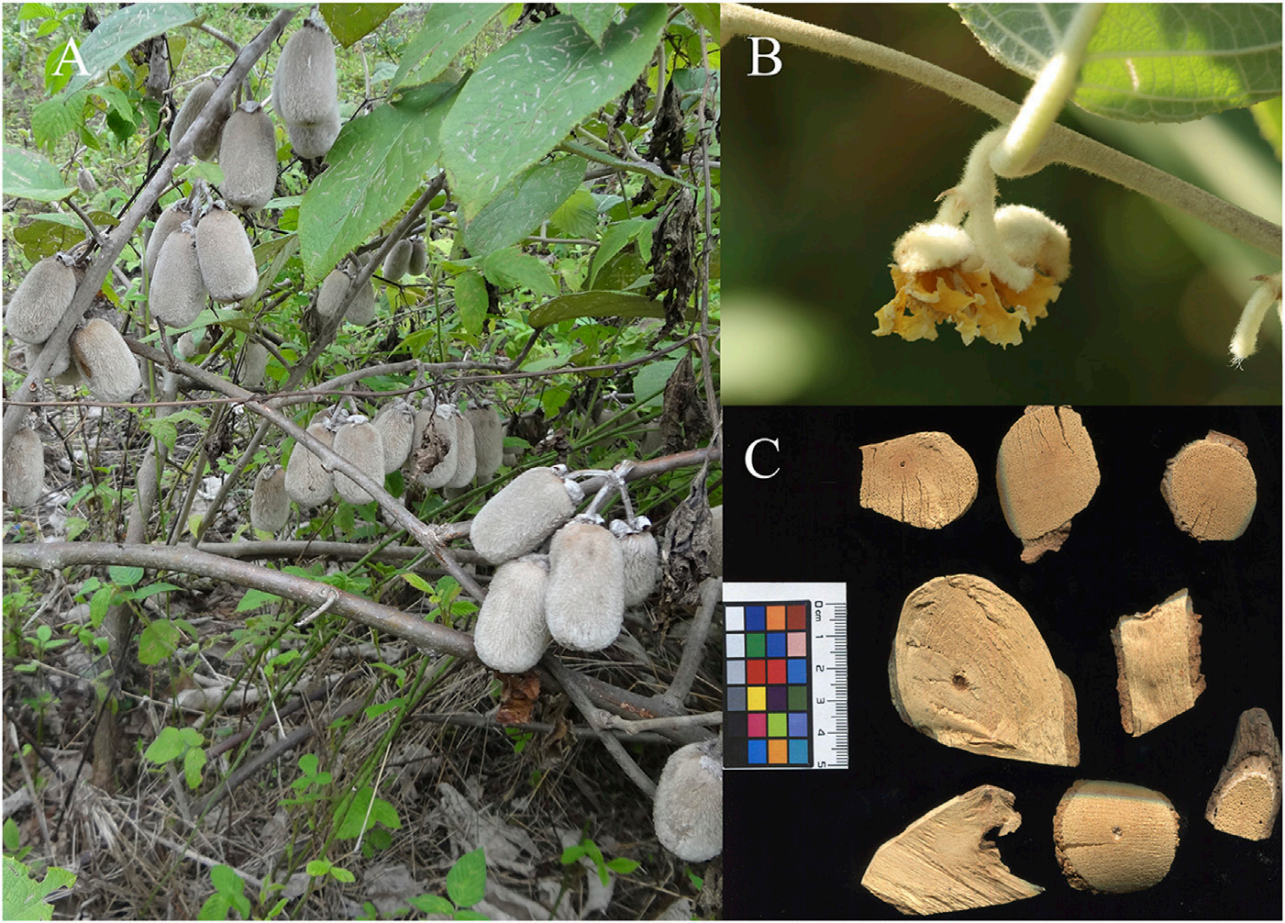
The whole plant **(A)**, flower portion **(B)**, and commercial herbal root pieces **(C)** of *A. eriantha.*

*Actinidia eriantha* (Figure 1) is a traditional medicine of *She* minority in China (Yu et al., 2017). As per “The Plant List” (www.theplantlist.org) database, *Actinidia eriantha* Benth. is the plant’s most accepted name. Other five synonyms for the plant are *Actinidia davidii* Franch, *Actinidia lanata* Hemsl., *Actinidia eriantha* f*. alba* C. F. Gan, *Actinidia eriantha* var. *brunnea* C. F. Liang and *Actinidia eriantha* var. *calvescens* C. F. Liang (www.theplantlist.org). AE is distributed in at least 13 provinces in China, with the main distribution area being the Yangtze River basin and high plant abundances being observed in Fujian, Zhejiang, and Jiangxi Provinces.

AE grows primarily at streamside or in forests at low altitudes (i.e., 200–1,200 m above sea level) but is also found at relative high altitudes (i.e., 1800–2000 m above sea level) on the Yunnan-Guizhou Plateau. AE has no specific requirements for soil conditions and can tolerate acidic soils with a pH value of 4.5–5.5. An average annual temperature of 9.2°C–17.4°C supports the normal growth and development of AE plants. However, the species can also tolerate harsh temperature conditions, for instance, a high temperature of 42.6°C and a low temperature of −27.4°C. Wild AE plants generally climb tall trees. Current season shoots of AE are grayish green in color with dense white short hairs, which are even more conspicuous at the tip; older shoots of AE, with or without Sun light exposure, are reddish-brown or dark gray in color, respectively. Other characteristics of AE shoots include lamellar whitish pith, 6–10-cm-long internodes, and a 6–8-mm diameter. The leaves of AE are single, alternate and can be pointed or elliptical in shape; the upper surface of AE leaves is glabrous with a dark green color, while the lower surface is gray green in color with dense white fluff. AE plants possess fleshy fibrous roots, 85% of which are distributed in the 0–40-cm-deep soil zone, while some can extend to around 60 cm below the soil surface. Most of AE roots are distributed in the 20–80-cm zone around the tree trunk, while some can reach 150 cm away from the trunk (Liao et al., 2021). The roots for medicinal use were collected in the autumn. The roots soaked to a certain extent are taken out and then watered until the internal and external humidity of the medicinal materials is consistent, thick slices, dried, and used for medicinal purpose (Zhejiang Food and Drug Administration, 2015).

## Phytochemistry

The phytochemical research on different AE organs, including root, leaf, and ripe fruit, has revealed the existence of various phytochemicals such as terpenoids, flavonoids glycoside, alcohols, aldehydes, ketones, organic acids, glucoside, and other compounds (Table 1). As far as the studies about the phytochemistry of AE are concerned, terpenoids that is the most deeply studied have been isolated and characterized from roots and aerial parts. What’s more, the phytochemicals extracted from root, the medicinal organ of AE, are even more worthy of further study. However, polysaccharides, the major type of bioactive compounds present in this herb (Guo et al., 2013), have not been isolated and still need further investigation. All in all, terpenoids and polysaccharides are the major biologically active constituents of AE.

**TABLE 1:** The chemical constituents isolated from *Actinidia eriantha* Benth.

Class	Compounds	Part of the plant	Chromatographic methods	Type of extract	References
Triterpenoid (26)	2α, 3β, 24-trihydroxy-urs-12-en-28-oic acid **1**	Aerial part	Slica column chromatography, thin layer chromatography	Methanolic extract	Bai et al. (1997a)
	2α, 3α, 24-trihydroxy-urs-12-en-28-oic acid **2**	Root, aerial part			Bai et al. (1997a), Huang et al. (1986), Zhang et al. (2017), Yu et al. (2020)
	2β, 3β-dihydroxy-23-oxo-urs-12-en-28-oic acid **3**	Root	High performance liquid chromatography, Slica column chromatography, thin layer chromatography	Ethyl acetate extract	Bai et al. (1997b), Guo, (2013)
	2α, 3α-dihydroxy-23-oxo-urs-12-en-28-oic acid **4**	Root	High performance liquid chromatography, thin layer chromatography	Chloroform extract	Guo (2013)
	2α, 3β, 23-trihydroxy-urs-12-en-28-oic acid **5**	Root	Slica column chromatography, thin layer chromatography	Methanolic extract	Bai et al. (1997b)
	2α, 3β-dihydroxy-12-en-28-oic acid **6**	Root	Slica column chromatography, thin layer chromatography	Ethyl acetate extract	Bai et al. (1997b)
	2α, 3α- dihydroxy-23-Garcia et al. (2012)methoxy-12-en-28-oic acid **7**	Root	High performance liquid chromatography, thin layer chromatography	Chloroform extract	Guo, (2013)
	3β,23,24-trihydroxyl-12-oleanen-28-oic acid **8**	Root	Gel colum chromatography, Sephadex LH-20 chromatography	Ethyl acetate extract	Wu et al. (2017)
	β-sitosterol **9**	Root, Aerial part	Slica column chromatography, thin layer chromatography		Bai et al. (1997a), Huang et al. (1986), Huang et al. (1988)
	Daucosterol **10**	Root, Aerial part	Slica column chromatography, thin layer chromatography		Bai et al. (1997a), Huang et al. (1986), Huang et al. (1988)
	ursolic acid **11**	Root, Aerial part	Slica column chromatography, thin layer chromatography		Bai et al. (1997a), Huang et al. (1988)
	Eriantic acid A **12**	Root	Slica column chromatography, thin layer chromatography		Huang et al. (1986)
	Eriantic acid B **13**	Root	Slica column chromatography, thin layer chromatography	Methanolic extract	Huang et al. (1988)
	Prenol **14**	Ripe fruit	Gas chromatography-mass spectrometry	Pentane and ether extract (with cyclohexanone as internal standard, and linalool as representative compound while standard was not available)	Garcia et al. (2012)
	*cis*-Linalool oxide **15**	Ripe fruit			Garcia et al. (2012)
	*trans*-Linalool oxide **16**	Ripe fruit			Garcia et al. (2012)
	Linalool **17**	Ripe fruit			Garcia et al. (2012)
	α-Terpineol **18**	Ripe fruit			Garcia et al. (2012)
	Nerol **19**	Ripe fruit			Garcia et al. (2012)
	*p*-Cymen-8-ol **20**	Ripe fruit			Garcia et al. (2012)
	2,6-Dimethyl-3,7-octadiene-2,6-diol **21**	Ripe fruit			Garcia et al. (2012)
	6,7-Dimethyl-7-hydroxylinalool **22**	Ripe fruit			Garcia et al. (2012)
	Perilla alcohol **23**	Ripe fruit			Garcia et al. (2012)
	(*Z*)-8- Hydroxylinalool **24**	Ripe fruit			Garcia et al. (2012)
	(*E*)-8- Hydroxylinalool **25**	Ripe fruit			Garcia et al. (2012)
	Geranic acid **26**	Ripe fruit			Garcia et al. (2012)
Alcohols (28)	2-Butanol **27**	Ripe fruit	Gas chromatography-mass spectrometry	Pentane and ether extract (with cyclohexanone as internal standard, and 1-hexanol as representative compound while standard was not available)	Garcia et al. (2012)
	2-Methyl-3-buten-2-ol **28**	Ripe fruit			Garcia et al. (2012)
	Isobutanol **29**	Ripe fruit			Garcia et al. (2012)
	3-Pentanol **30**	Ripe fruit			Garcia et al. (2012)
	2-Pentanol **31**	Ripe fruit			Garcia et al. (2012)
	Butanol **32**	Ripe fruit			Garcia et al. (2012)
	1-Penten-3-ol **33**	Ripe fruit			Garcia et al. (2012)
	4-Methyl-2-pentanol **34**	Ripe fruit			Garcia et al. (2012)
	3-Hexanol **35**	Ripe fruit			Garcia et al. (2012)
	Isoamyl alcohol **36**	Ripe fruit			Garcia et al. (2012)
	3-Methyl-3-buten-1-ol **37**	Ripe fruit			Garcia et al. (2012)
	Cyclopentanol **38**	Ripe fruit			Garcia et al. (2012)
	3-Methyl-1-pentanol **39**	Ripe fruit			Garcia et al. (2012)
	Hexanol **40**	Ripe fruit			Garcia et al. (2012)
	(*Z*)-3-Hexen-1-ol **41**	Ripe fruit			Garcia et al. (2012)
	3-Octanol **42**	Ripe fruit			Garcia et al. (2012)
	(*E*)-2-Hexen-1-ol **43**	Ripe fruit			Garcia et al. (2012)
	Cyclopentanemethanol **44**	Ripe fruit			Garcia et al. (2012)
	1-Octen-3-ol **45**	Ripe fruit			Garcia et al. (2012)
	Sulcatol **46**	Ripe fruit			Garcia et al. (2012)
	2-Ethyl-1-hexanol **47**	Ripe fruit			Garcia et al. (2012)
	1,2-Pentanediol **48**	Ripe fruit			Garcia et al. (2012)
	7-Methyl-4-octanol **49**	Ripe fruit			Garcia et al. (2012)
	1,3-Octanediol **50**	Ripe fruit			Garcia et al. (2012)
	Benzyl alcohol **51**	Ripe fruit			Garcia et al. (2012)
	2-Phenyl-1-ethanol **52**	Ripe fruit			Garcia et al. (2012)
	2,4-Dimethylphenethyl alcohol **53**	Ripe fruit			Garcia et al. (2012)
	*o*-Methoxybenzyl alcohol **54**	Ripe fruit			Garcia et al. (2012)
Phenolics (15)	Methyl salicylate **55**	Ripe fruit	Gas chromatography-mass spectrometry	Pentane and ether extract (with cyclohexanone as internal standard, and vanillin as representative compound while standard was not available)	Garcia et al. (2012)
	Phenol **56**	Ripe fruit			Garcia et al. (2012)
	*p*-Cresol **57**	Ripe fruit			Garcia et al. (2012)
	Eugenol **58**	Ripe fruit			Garcia et al. (2012)
	4-Vinylguaiacol **59**	Ripe fruit			Garcia et al. (2012)
	4-Hydroxy-3-methylacetophenone **60**	Ripe fruit			Garcia et al. (2012)
	*cis*-Isoeugenol **61**	Ripe fruit			Garcia et al. (2012)
	(*E*)-Cinnamyl alcohol **62**	Ripe fruit			Garcia et al. (2012)
	*trans-*Isoeugenol **63**	Ripe fruit			Garcia et al. (2012)
	Vanillin **64**	Ripe fruit			Garcia et al. (2012)
	Homovanillic acid **65**	Ripe fruit			Garcia et al. (2012)
	*p*-Hydroxyphenethyl alcohol **66**	Ripe fruit			Garcia et al. (2012)
	3,4,5-Trimethoxyphenol **67**	Ripe fruit			Garcia et al. (2012)
	Coniferyl alcohol **68**	Ripe fruit			Garcia et al. (2012)
	Benzaldehyde **69**	Ripe fruit			Garcia et al. (2012)
Aldehydes (2)	Hexanal **70**	Ripe fruit	Gas chromatography-mass spectrometry	Pentane and ether extract (with cyclohexanone as internal standard, and (*E*)-2-hexenal as representative compound while standard was not available)	Garcia et al. (2012)
	(*E*)-2-Hexenal **71**	Ripe fruit			Garcia et al. (2012)
Organic acid (14)	Acetic acid **72**	Ripe fruit	Gas chromatography-mass spectrometry	Pentane and ether extract (with cyclohexanone as internal standard, and acetic acid as representative compound while standard was not available)	Garcia et al. (2012)
	Isobutyric acid **73**	Ripe fruit			Garcia et al. (2012)
	Isovaleric acid **74**	Ripe fruit			Garcia et al. (2012)
	2-Ethylbutanoic acid **75**	Ripe fruit			Garcia et al. (2012)
	Hexanoic acid **76**	Ripe fruit			Garcia et al. (2012)
	*trans-*2-Hexenoic acid **77**	Ripe fruit			Garcia et al. (2012)
	Octanoic acid **78**	Ripe fruit			Garcia et al. (2012)
	Nonanoic acid **79**	Ripe fruit			Garcia et al. (2012)
	Hexadecanoic acid **80**	Ripe fruit			Garcia et al. (2012)
	Octadecanoic acid **81**	Ripe fruit			Garcia et al. (2012)
	Linoletic acid **82**	Ripe fruit			Garcia et al. (2012)
	Linolenic acid **83**	Ripe fruit			Garcia et al. (2012)
	Benzoic acid **84**	Ripe fruit			Garcia et al. (2012)
	Phenylacetic acid **85**	Ripe fruit			Garcia et al. (2012)
Flavonoids glycoside (8)	Isorhamnetin 3-O-rhamnose 1-6-glucose **86**	Leave	Paper chromatography, Column chromatography, thin layer chromatography	Ethanolic extract	Webby et al. (1994)
	Kaempferol 3-O-rhamnose 1-6-glucose **87**	Leave			Webby et al. (1994)
	Quercetin 3,7-O-di, tri glycoside **88**	Leave			Webby et al. (1994)
	Quercetin 3-O-rhamnose rhamnose glucose **89**	Leave			Webby et al. (1994)
	Kaempferol 3-O-rhamnose 1–4 rhamnose 1–6 glucose **90**	Leave			Webby et al. (1994)
	Quercetin 3-O- glucose **91**	Leave			Webby et al. (1994)
	Kaempferol 3-O- glucose **92**	Leave			Webby et al. (1994)
	Quercetin 3-O-rhamnose 1–6 galactose **93**	Leave			Webby et al. (1994)
Ketones (4)	(*E*)-3-Penten-2-one **94**	Ripe fruit	Gas chromatography-mass spectrometry	Pentane and ether extract (with cyclohexanone as internal standard, and β-damascenone as representative compound while standard was not available)	Garcia et al. (2012)
	4-Methyl-3-penten-2-one **95**	Ripe fruit			Garcia et al. (2012)
	4-Hydroxy-4-methyl-2- pentanone **96**	Ripe fruit			Garcia et al. (2012)
	4-Hydroxy-5-methyl-2-hexanone **97**	Ripe fruit			Garcia et al. (2012)
Glucoside (2)	(6R,7E,9S)-6,9-hydroxy-megastiman-4,7-dieu-3-one-9-O-β-D-glucopyranoside **98**	Root	Gel colum chromatography, Sephadex LH-20 chromatography	Ethyl acetate extract	Wu et al. (2017)
	Oleanolic acid-23-O-β-D- glucopyranoside **99**	Root			Wu et al. (2017)
Others (5)	Dihydro-3,5-dimethyl-2(3*H*)-furanone **100**	Ripe fruit	Gas chromatography-mass spectrometry	Pentane and ether extract (with cyclohexanone as internal standard, and β-damascenone as representative compound while standard was not available)	Garcia et al. (2012)
	Furfuryl alcohol **101**	Ripe fruit			Garcia et al. (2012)
	3-Hydroxy-b-damascone **102**	Ripe fruit			Garcia et al. (2012)
	2-(Methylthio)ethanol **103**	Ripe fruit			Garcia et al. (2012)
	3-(Methylthio)-1-propanol **104**	Ripe fruit			Garcia et al. (2012)

### Triterpenoids

**FIGURE 2:**
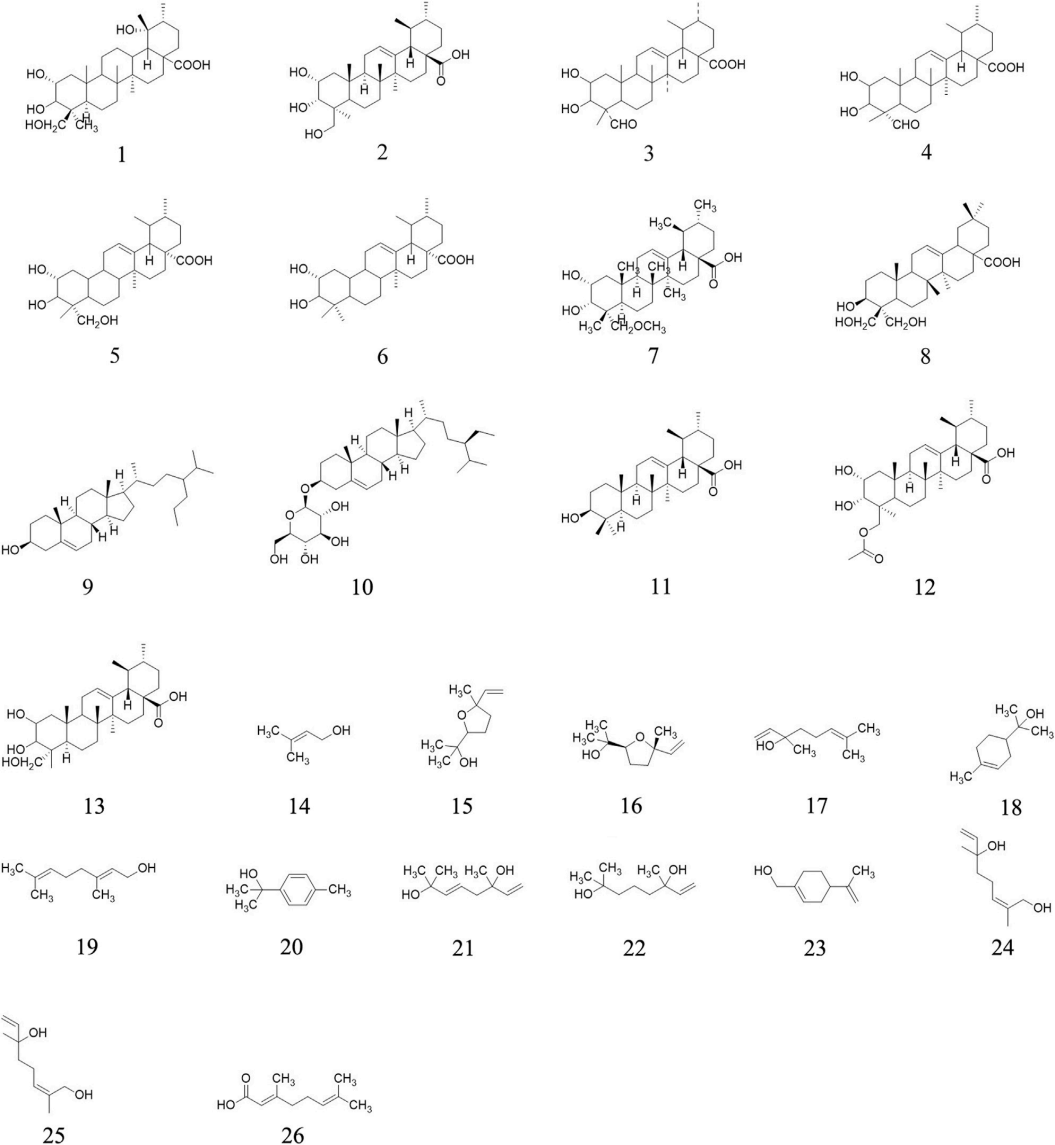
Triterpenoids isolated or determined from *A. eriantha*.

Triterpenoids represent the main bioactive substances in AE. Totally, twenty-nine triterpenoids have been isolated from AE, including 2α, 3β, 24-trihydroxy-urs-12-en-28-oic acid **1** (Bai et al., 1997a), 2α,3α,24-trihydroxy-urs-12-en-28-oic acid **2** (Huang et al., 1986; Huang et al., 1988; Bai et al., 1997a; Zhang et al., 2017; Yu et al., 2020), 2β, 3β-dihydroxy-23-oxo-urs-12-en-28-oic acid **3** (Bai et al., 1997a), 2α,3α-dihydroxy-23-oxo-urs-12-en-28-oic acid **4** (Guo, 2013), 2α, 3β, 23-trihydroxy-urs-12-en-28-oic acid **5** (Bai et al., 1997b), 2α, 3β-dihydroxy-12-en-28-oic acid **6** (Bai et al., 1997b), 2α,3α-dihydroxy-23-methoxy-12-en-28-oic acid **7** (Guo, 2013), 3β,23,24-trihydroxyl-12-oleanen-28-oic acid **8** (Wu et al., 2017) β-sitosterol **9** (Huang et al., 1986; Huang et al., 1988; Bai et al., 1997a), daucosterol **10** (Huang et al., 1986; Huang et al., 1988; Bai et al., 1997a), etc. Triterpenoids are isolated from root and ripe fruits. However, the pharmacological effects of terpenoids are yet to be well defined. The chemical structures of the triterpenoids are displayed in Figure 2.

### Alcohols

**FIGURE 3:**
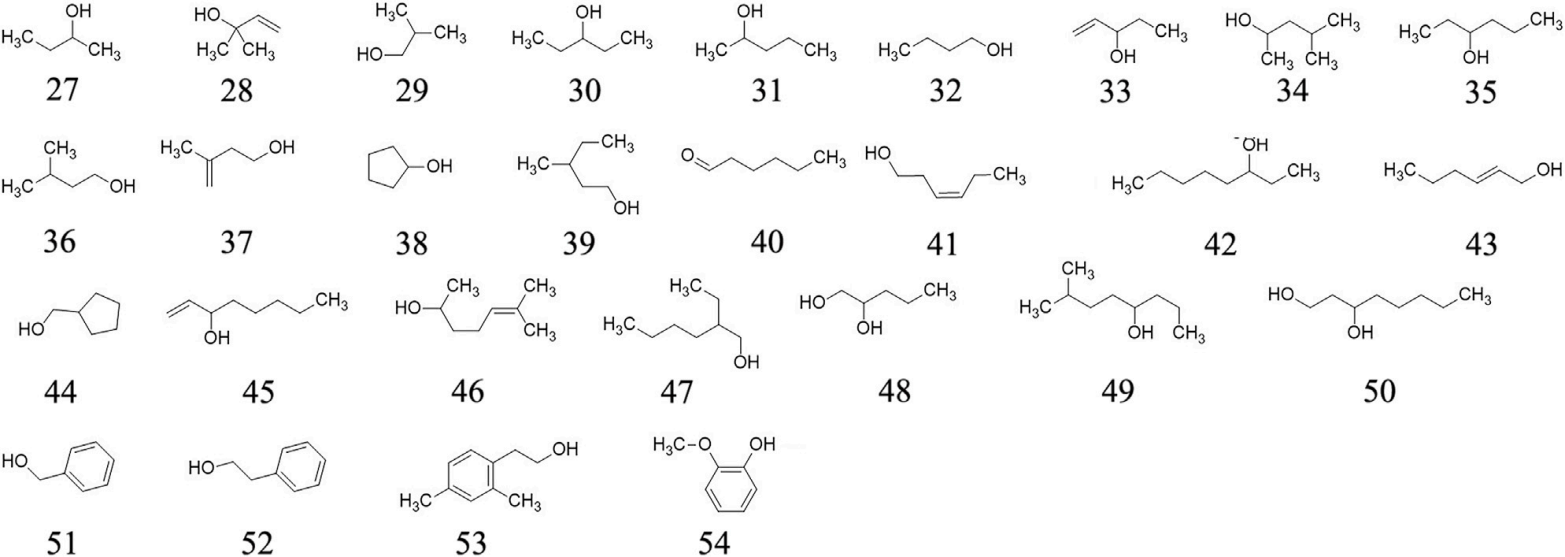
Alcohols determined from *A. eriantha*.

Twenty-four alcohols are determined in ripe fruits of AE plants through GC–MS, including 2-Butanol **27**, 2-Methyl-3-buten-2-ol **28**, Isobutanol **29**, 3-Pentanol **30**, 2-Pentanol **31**, Butanol **32**, 1-Penten-3-ol **33**, 4-Methyl-2-pentanol **34**, 3-Hexanol **35**, Isoamyl alcohol **36**, etc. (Garcia et al., 2012). These molecules’ chemical structures are presented in Figure 3.

### Phenolics and aldehydes

**FIGURE 4:**
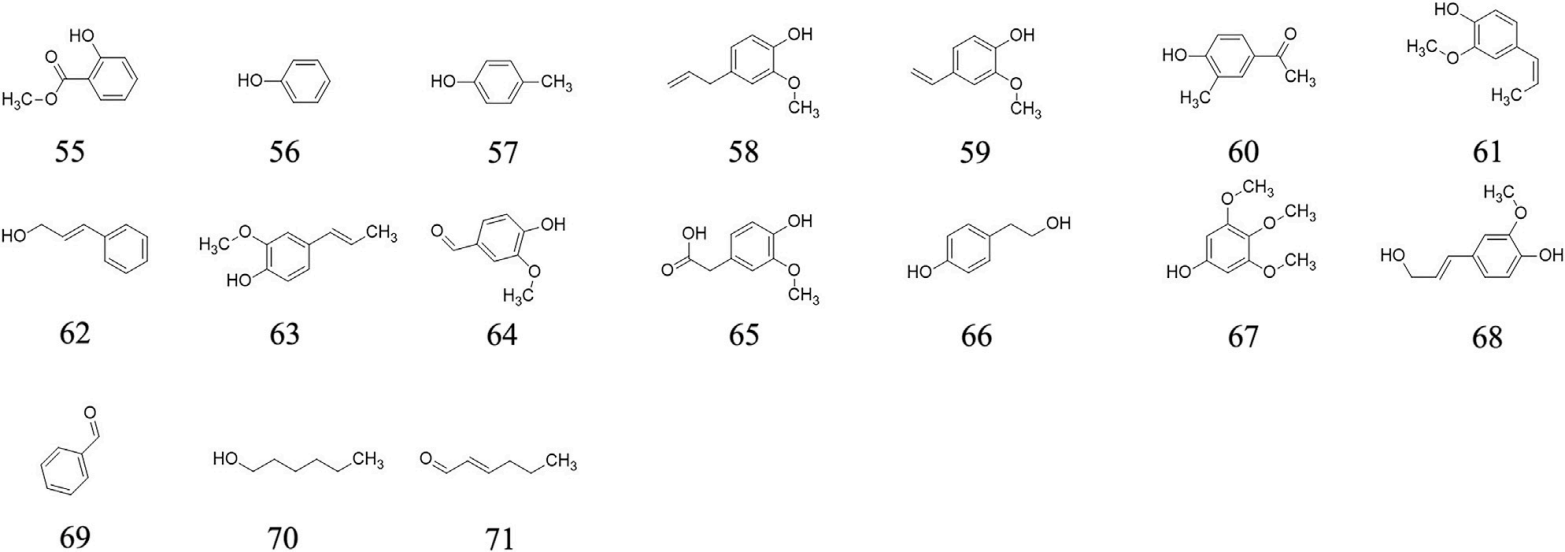
Phenolics and aldehydes determined from *A. eriantha*.

Fifteen phenolics (methyl salicylate **55**, phenol **56**, p-Cresol **57**, eugenol **58**, 4-Vinylguaiacol **59**, 4-Hydroxy-3-methylacetophenone **60**, cis-Isoeugenol **61**, (*E*)-Cinnamyl alcohol **62**, *trans*-Isoeugenol **63**, vanillin **64**, homovanillic acid **65**, *p*-Hydroxyphenethyl alcohol **66**, 3,4,5-Trimethoxyphenol **67**, coniferyl alcohol **68,** Benzaldehyde **69**) and two aldehydes (hexanal **70**, (E)-2-Hexenal **71**) are determined from AE ripe fruit by GC-MS (Garcia et al., 2012). The chemical structures of the phenolics and aldehydes are exhibited in Figure 4.

### Organic acids

**FIGURE 5:**
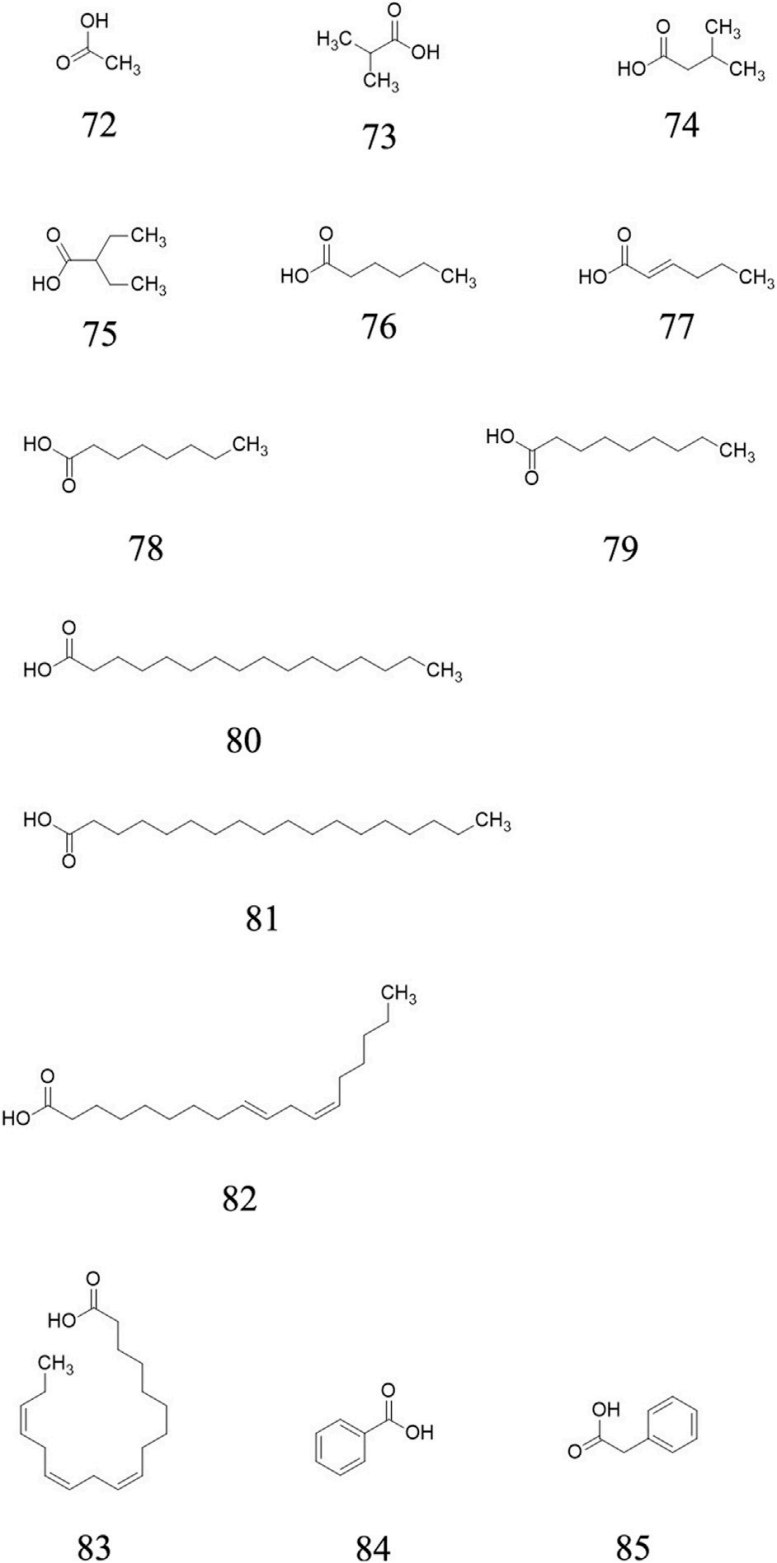
Organic acid determined from *A. eriantha*.

Twelve acid, including acetic acid **72**, isobutyric acid **73**, isovaleric acid **74**, 2-Ethylbutanoic acid **75**, hexanoic acid **76**, *trans*-2-Hexenoic acid **77**, octanoic acid **78**, nonanoic acid **79**, hexadecanoic acid **80**, octadecanoic acid **81**, linoletic acid **82**, linolenic acid **83**, benzoic acid **84** and phenylacetic acid **85** are confirmed from the ripe fruits of AE plants (Garcia et al., 2012). Their chemical structures are presented in Figure 5.

### Flavonoids glycoside

Eight flavonoids (isorhamnetin 3-O-rhamnose 1-6-glucose **86**, kaempferol 3-O-rhamnose 1-6-glucose **87**, quercetin 3,7-O-di, tri glycoside **88**, quercetin 3-O-rhamnose rhamnose glucose **89**, kaempferol 3-O-rhamnose 1-4 rhamnose 1-6 glucose **90**, quercetin 3-O- glucose **91**, kaempferol 3-O- glucose **92**, quercetin 3-O-rhamnose 1-6 galactose **93**) are isolated from the leaf of AE plants (Webby et al., 1994).

### Ketones

**FIGURE 6:**
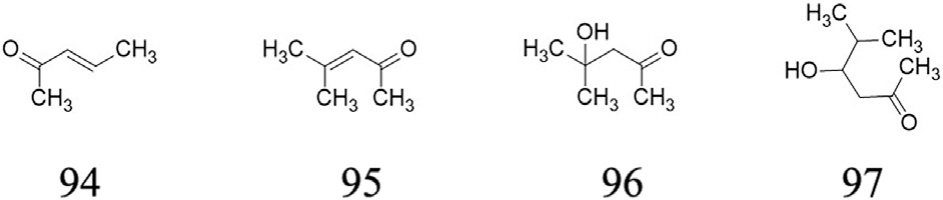
Ketones determined from *A. eriantha*.

Four ketones ((E)-3-Penten-2-one **94**, 4-Methyl-3-penten-2-one **95**, 4-Hydroxy-4-methyl-2- pentanone **96**, 4-Hydroxy-5-methyl-2-hexanone **97**) are determined from ripe fruits from AE plants (Garcia et al., 2012). These substances’ chemical structures are displayed in Figure 6.

### Glucoside and others

**FIGURE 7:**
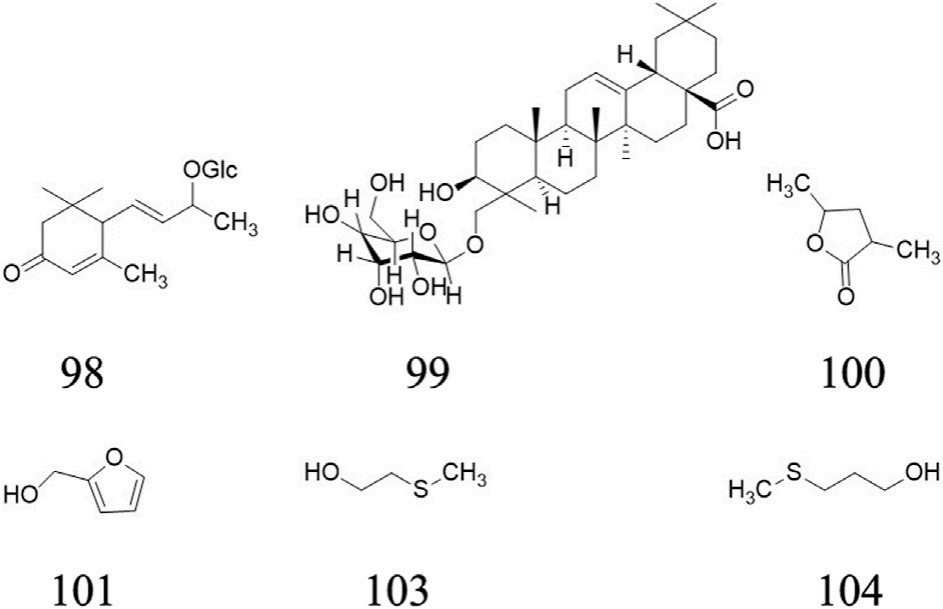
Glucoside, and others determined from *A. eriantha*.

Two Glucoside, (6R,7E,9S)-6,9-hydroxy-megastiman-4,7-dieu-3-one-9-O-β-D-glucopyranoside **98** (Wu et al., 2017), Oleanolic acid-23-O-β-D- glucopyranoside **99** (Wu et al., 2017) and two furans (Dihydro-3,5-dimethyl-2(3H)-furanone **100** (Garcia et al., 2012), Furfuryl alcohol **101** (Garcia et al., 2012)as well as other compounds, such as 3-Hydroxy-b-damascone **102** (Garcia et al., 2012), 2-(Methylthio)ethanol **103** (Garcia et al., 2012), 3-(Methylthio)-1-propanol **104** (Garcia et al., 2012), polysaccharide AEPS (Xu et al., 2009b; Chen et al., 2019b), are determined and isolated from roots and ripe fruits of AE plants. These compounds’ chemical structures are exhibited in Figure 7.

## Pharmacological activities

**FIGURE 8:**
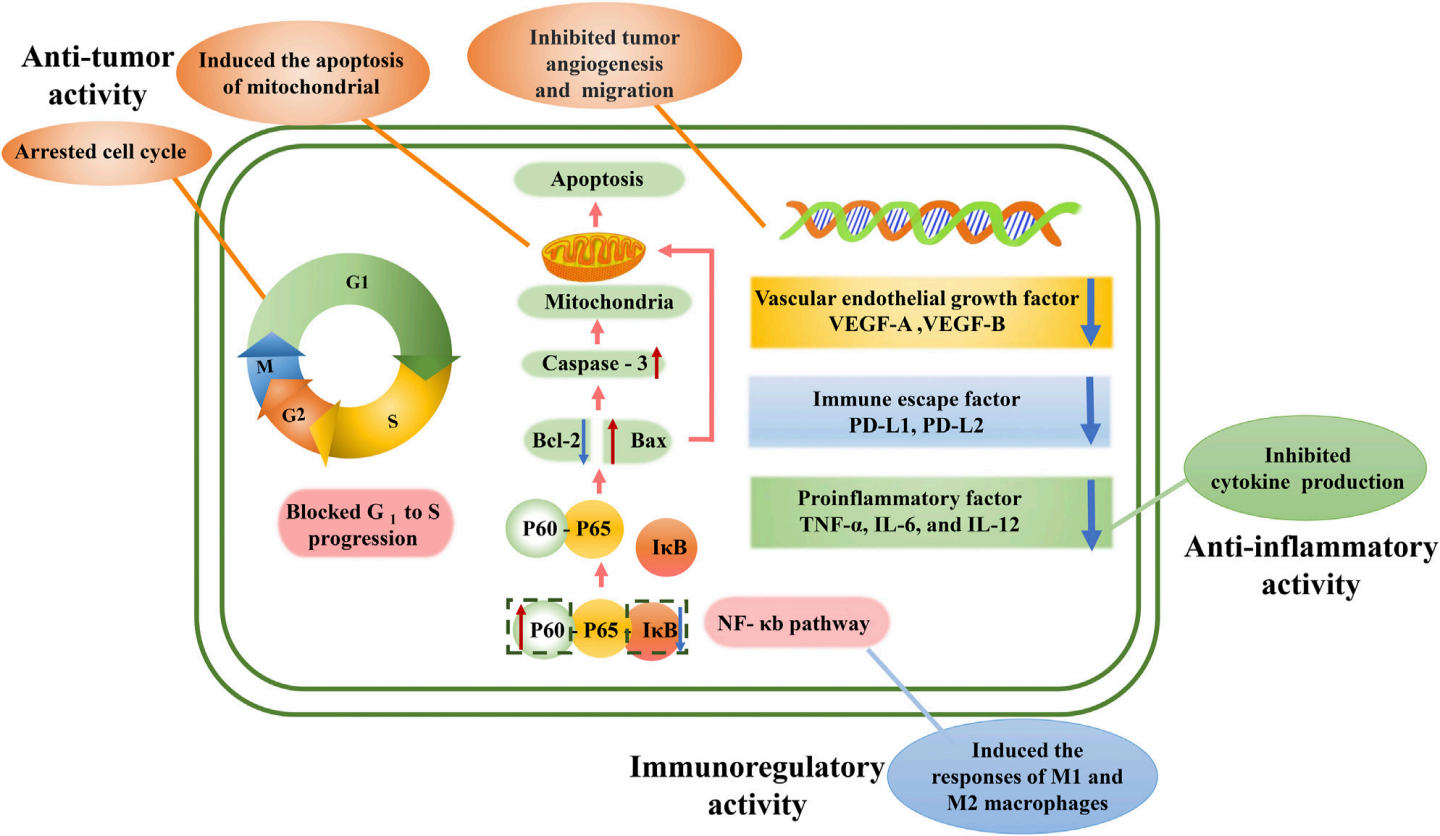
Possible mechanism of anti-tumor, immunoregulatory, and anti-inflammatory activities of *A. eriantha*. S, Synthesis phase, G, Gap phase, M, mitotic period. AE inhibited tumor development through arresting cell cycle, inducing apoptosis and inhibiting tumor angiogenesis and migration. AE regulated immunity through inducing the responses of M1 and M2 macrophages. AE exhibited anti-inflammatory activity through inhibiting cytokine production.

AE has been demonstrated to possess various pharmacological activities. The main pharmacological activities are anti-tumor activity, as well as other activities such as immunoregulatory, anti-inflammatory, anti-angiogenic, and neuroprotective and antioxidant activities. As a traditional medicine in *She* ethnic minority group, a minority who lived in Zhejiang and Fujian Provinces, AE is widely used to treat stomach cancer, colon cancer, cirrhosis with ascites, chronic hepatitis, leukemia, rectal prolapse, hernia and uterine prolapse. However, pharmacological studies only provide evidence for the traditional use of its anti-tumor effect, while other activities such as treating chronic hepatitis still need further research. The possible mechanism of anti-tumor, immunoregulatory, and anti-inflammatory activities are shown in Figure 8. And these activities have been displayed in Table 2 and will be discussed further in the following sections.

**TABLE 2:** Pharmacological activities of *Actinidia eriantha* Benth.

Pharmacological activity	Tested substance	Model	Tested living system/organ/cell	Result	Dose range	Time period of application	References
Immunoregulatory activity	Aeps	Raw264.7cells	Cells	Induced the activation of macrophages via lncrnas/nf-κb networks	50 μg/ml	4 h	Chen et al. (2019a)
	AEPS	RAW264.7cells	Cells	Induced macrophage activation through regulating mirnas expression	50 μg/ml	24 h	Chen et al. (2019b)
	AEPS	Mice	Supernatant and the elleted cells	Induced the expression of large numbers of cytokines and chemokines	50 μg	3, 6 h	Du et al. (2020)
	AEPS	RAW264.7 cells	Cells	Enhanced the pinocytic and phagocytic activity, promote the expression of accessory and costimulatory molecules	0–200 μg/ml	24 h	Sun et al. (2015)
	AEPS	Icrmice	Sera and splenocyte	Increased both cellular and humoral immune responses and elicited a balanced Th1/Th2 response	25,50, 100 μg	2 weeks	Sun et al. (2009)
	Phenoplic extracts	Splenocyte	Cell	Induced the proliferation and reduced IFN-γ production	62.5–1,000 μg/ml	48 h	Kim et al. (2018)
Antitumor activity	Eel, ees	Huvecs	Cells	Decreased the Cell viability	100 μg/ml	24 h	Wu et al. (2017)
	EER	SGC7901 cells, CNE2 cells and huvecs	Cells	Inhibited the cells’ growth	100 μg/ml	24 h	Wu et al. (2017)
	PE-EER, BA-EER, WE-EER	Huvecs	Cells	Inhibited the cells’ growth	100 μg/ml	24 h	Wu et al. (2017)
	EA-EER	CNE2 cells	Cells	Inhibited the cells’ growth	100 μg/ml	24 h	Wu et al. (2017)
	EA-EER	SGC7901 cells, huvecs	Cells	Inhibited the cells’ growth in a time and dose-dependent manner	0–100 μg/ml	24, 48, 72 h	Wu et al. (2017)
	EA-EER	SGC7901 cells	Cells	Decreased the Number of cells and ncreasing degree of apoptosis with some obvious apoptotic morphological alterations	0, 50, 75 and 100 μg/ml	24 h	Wu et al. (2017)
	EA-EER	Huvecs	Cells	Induced apoptosis	0, 40, 60, 80 μg/ml	24 h	Wu et al. (2017)
	EA-EER	Huvecs	Cells	Inhibit cell migration of huvecs in a dose-dependent manner	0, 30, 40, 60 μg/ml	24 h	Wu et al. (2017)
	EA-EER	Chick CAM model	Blood vessels	Was capable of restraining angiogenesis *in vivo*	1.0 mg/ml	72 h	Wu et al. (2017)
	AEPS and AEPA, AEPB, AEPC, AEPD	Tumor-bearing mice	Tumors	Inhibited the growth of tumor transplanted	2.5, 5.0, 10.0 mg/kg	10 days	Xu et al. (2009b)
	AEPS and AEPA, AEPB, AEPC, AEPD	S180-bearing mice	S180 sarcoma	Inhibited the growth of transplantable S180 sarcoma in mice and promoted splenocytes proliferation, natural killer cells activity, interleukin-2 production from splenocytes and serum tumor antigen-specific antibody levels in tumor-bearing	10 mg/kg	5 days	Xu et al. (2009a)
Anti-angiogenic activity	Ea-eer	Sgc7901 cells	Cells	Downregulated mrna expression of bcl-2 and up-regulated mrna expression of bcl-2 and the protein expression of caspase-3in sgc7901 cells, in a dose-dependent manner	0, 40, 60, 80 μg/ml	24 h	Wu et al. (2017)
	EA-EER	Huvecs	Cells	Reduced mrna expression of VEGF-A and VEGFR-2 in huvecs	0, 40, 60, 80 μg/ml	24 h	Wu et al. (2017)
Neuroprotective activity	Aqueous ethanol	Pc-12 cells	Cells	Protected neuron-like pc-12 cells from aβ_1-42_ -induced neurotoxicity	62.5, 250, and 1,000 μg/ml	24 h	Cho et al. (2021)
	Aqueous ethanol	ICR mice	Mice	Prevented cognitive impairment	50, 200, and 1,000 mg/kg	3 weeks	Cho et al. (2021)
	Aqueous ethanol	ICR mice	Brain tissue	Ameliorated Aβ_1-42_ -induced spatial cognitive learning and memory deficits and protected the antioxidant defense systems in brain tissue	50, 200, and 1,000 mg/kg	3 weeks	Cho et al. (2021)
Anti-inflammatory activity	Phenolic extracts	Macrophages	Cells	Inhibited the production of the pro-inflammatory cytokines	62.5–1,000 μg/ml	48 h	Kim et al. (2018)

### Anti-tumor activity

Cancer is the second most common disease worldwide and is difficult to treat (Zhu et al., 2018). Investigations utilizing *in vivo* and *in vitro* systems have demonstrated that AE extracts, including ethanol extracts, n-BuOH extracts, ethyl acetate extracts, petroleum ether extract, aqueous extract, chloroform extract and methanol extract, possessed anti-tumor activity. The aqueous extracts of AE roots can not only inhibit the proliferation, migration and invasion of H1299 cells in dose-dependently manner *via* upregulated the PCDH10 gene expression by downregulating mir-182-5p gene expression (Zhao et al., 2020), but also inhibited M21 cell proliferation, invasion and migration by downregulating the expression of pD-L1 and PD-L2 molecular genes and proteins closely related to immune escape of tumor cells (Wang et al., 2013).

A 24 h administration of ethyl acetate fraction from the root of AE viz. EA-EER (100 μg/ml) significantly suppressed the proliferation of HUVECs by blocking G1 to S cell cycle progression and downregulating VEGF-A and VEGFR-2 expression (Wu et al., 2017). In addition, EA-EER inhibited Bcl-2 expression and enhanced Bax and caspase-3 expression in SGC7901 cells (Wu et al., 2017). These results suggested that EA-EER has the potential to serve as a source of anti-cancer drugs. However, it was necessary to explore the bioactivity of the compounds extracted and the trial lacked a positive control. Tang et al. confirmed the main active ingredient of EA-EER to be 2α,3α,24-trihydroxyurs-12-en-28-oic acid by HPLC, and found that its nano-micelles could inhibit the survival rate of U87MG cells in an administration of 0–80 μg/ml dose-dependently. After being loaded with nano-micelles, the compound exhibited better inhibiting effect on U87MG cells than monomeric compound, while the enhanced effect can be attributed to nano-micelles’ improving water solubility of compound (Tang et al., 2019). Nevertheless, the administration of chloroform extract from the AE roots (4.5–450 μg/ml) dose-dependently suppressed SMMC-7721 cell proliferation (Wang et al., 2011). In another study, administration of chloroform extract from the AE roots (87.5–200 μg/ml) also dose-dependently suppressed BEL-7404 cell proliferation and it was considered that the active components are alkaloids (Guo et al., 2013).

Xu et al. (2009b) has found that the administration of total polysaccharides (AEPS) and four polysaccharides (namely AEPA-AEPD) extracted from AE roots (10 mg/kg) could suppress the growth of S180 sarcoma or H22 hepatoma xenografts in mice. However, the research lacked a positive control for the tumor-suppressing effects of the polysaccharides from AE.

### Immunoregulatory activity

Spleen, as an essential peripheral lymphatic organ, it key for innate immunological responses because it can participate in phagocytosis and immune memory (Zhu et al., 2018). In the models of OVA-immunized mice and OVA-induced splenocytes, the administration of AEPS (62.5–1,000 μg/ml) significantly promoted the proliferation of splenocytes treated with Con A and LPS and reduced the production of INF-c relative to the splenocytes stimulated by an anti-CD3 mAb (Kim et al., 2018). However, there was no positive control for that study, and an analysis of dose-effect relationship was also lacking, which had negatively affected the reliability of the study. In another study, administration of AEPA-AEPD could significantly enhance the proliferation of splenocytes, increase the activity of natural killer (NK) cells, stimulate the secretion of interleukin-2 by splenocytes and upregulate the level of tumor antigen-specific antibody in the serum of tumor-bearing mice, which suggested that the anti-tumor activity of AEPA-AEPD was realized by enhancing immune response (Xu et al., 2009a). Furthermore, administration of AEPS (25, 50, or 100 μg) and OVA for 2 weeks significantly increased the serum level of OVA-specific antibody, the cytotoxic activity of NK cells, and the expression and secretion of Th1/Th2 cytokines by activating related transcription factors in the splenocytes of mice treated with OVA (Sun et al., 2009). In OVA induced BALB/c mice, the administration of AEPS triggered an immune effector process manifesting as monocyte, dendritic cell, and neutrophil recruitment and higher expression levels of CXCL2, CXCL3, CXCL5, CXCL10, CCL2, CCL3, CCL4, CCL7, IL-12β, and IL-23α mRNA (Du et al., 2020). Nevertheless, a 24-h administration of AEPS (10 mg/ml) resulted in the differential expression of 82 miRNAs in RAW264.7 cells, among which 43 and 39 were up- and downregulated, respectively (Chen et al., 2019a). In in vitro studies on RAW264.7 cells, AEPS evoked the responses of M1 and M2 macrophages *via* the NF-κB pathway (Sun et al., 2015; Chen et al., 2019a). These findings reflected that AEPS might have induced macrophage activation by regulating the expression of miRNAs and the activity of the NF-κB pathway. However, it is necessary to carry out further *in vivo* studies to investigate the physicochemical characteristics of these extracts and their mechanisms of action from the perspective of intracellular signaling pathways. In addition, there existed robust patterns of lncRNAs expression corresponding to the specific adjuvants used during the biological processes mentioned above, implying that lncRNAs are involved in the immune responses stimulated by AEPS(Du et al., 2020). This research shed more light on the adjuvants’ molecular mechanisms and provided guidance for the reasonable design of vaccines with high efficacy. However, other factors should also be considered when making conclusive decisions on the predictor organism.

### Anti-inflammatory activity

The overproduction of pro-inflammatory factors, such as TNF-α and IL-6, has been associated with several inflammatory disorders, including rheumatoid arthritis, Alzheimer’s disease, and cancer^7^. It was found that the phenolic extracts from AE kiwifruit could decrease the concentrations of TNF-α, IL-6, and IL-12 in the cell culture medium for primary macrophages obtained from male BALB/c mice (Kim et al., 2018). Moreover, the phenolic extracts significantly attenuated INF-γ production in splenocytes. However, there is a need to investigate the chronic toxicity of AE and identified the isolated activity compounds (Kim et al., 2018).

### Other activities

In classic chick CAM model, the treatment of EA-EER profoundly decreased the number of blood vessels relative to the untreated control, indicating that EA-EER could restrain angiogenesis *in vivo* (Wu et al., 2017). In addition, the Aβ 1–42 -treated ICR mice experiment showed that AEE (*A. eriantha* cv. Bidan extract) at doses of 50, 200, and 1,000 mg/kg body weight per day could alleviate learning and spatial memory deficiencies and activate intracellular antioxidant systems (CAT, SOD, and GSH/GSSG) in brain tissues of mice. These results indicated that the mechanisms underlying the cell protection effects of AEE include the inhibition of apoptosis-related signaling pathways and the protection of mitochondra (Cho et al., 2021). Furthermore, AE also exhibited strong antioxidant capacity with high ferric reducing ability of plasma (FRAP) value and ·O^2−^ clearance rate (Huang et al., 2020). The antioxidant capacities of AE kiwifruit were determined to be 608.9, 620.9 and 1,016.8 mg VCE/100 g fresh weight, respectively, by the ABTS, DPPH and ORAC experiments (Kim et al., 2018). However, many traditional uses of AE have not been the focus of recent research. For example, although many traditional documents have revealed that AE can benefit cirrhosis with ascites, chronic hepatitis, leukemia, rectal prolapse, hernia and uterine prolapse, these beneficial effects themselves and the mechanisms underlying them have not been widely investigated. Therefore, these traditional uses of AE warrant further investigations.

## Traditional uses

The genus *Actinidia* was first recorded in Book of Songs (《诗经》) which dates to the Xizhou and Chunqiu period (B. C. 1,000–600). *Actinidia* has been widely used in China as both functional foods (Fruit) and medicines (Root). Root of *Actinidia* is recorded in many ancient herbal texts, such as Supplement to Herbology (《本草拾遗》) (Tang Dynasty, A. D. 739), Kaibao Herbology (《开宝本草》) (Song Dynasty, A. D. 973–974), and Compendium of Materia Medica (《本草纲目》) (Ming Dynasty, A. D. 1,578), and is widely used to treat arthrosis pain, traumatic injury, haemorrhoids, filariasis, dysentery, and pyogenic infections (Di et al., 2014). However, it is still not sure when the root of *Actinidia* was distinguished into *Actinidia Chinensis* (one sibling species of AE) root and AE root.

AE has many folk names, including *Baishanmaotaogen*, *Baimaotao*, *Maohuayangtao*, *Baitengli*, *Tengligen* (Lei and Li 2007; Zhu et al., 2015). In TCM, AE is used to treat stomach cancer, colon cancer, cirrhosis with ascites, chronic hepatitis, leukemia, rectal prolapse, hernia and uterine prolapse (Zhu et al., 2015). AE roots has been used in many *She* ethnic minority group traditional preparations. AE roots is often used in combination with *Scutellaria barbata*, *Hedyotis diffusa* and *Rabdosia amethystoides* to treat tumors, colon cancer, cirrhosis of the liver, and leukemia. In addition to this, it also used in combination with *Aralia chinensis* roots and *Ampelopsis sinica* to treat fracture and sprain. Examples of traditional Chinese medicine prescriptions containing AE are listed in the Table 3. However, the potential interactions and synergistic effects between the active components of AE and those of other herbal medicines, as well as the underlying mechanism of action, have not been clarified and warrant further investigation.

**TABLE 3:** Examples of traditional Chinese medicine prescriptions containing *Actinidia eriantha* Benth.

Traditional and clinical uses	Prescription composition	Role of AE in prescription	References
Treat hernia, rectocele, and orchitis	*Actinidia eriantha*, *Fortunella hindsii*	Leading role	Chen and Guan (1996), Lei and Li (2007)
Treat leukemia	*Actinidia eriantha*, *Scutellaria barbata*, *Hedyotis diffusa, Cephalotaxus fortune*, *Rabdosia amethystoides*, *Ampelopsis sinica*	Leading role	Lei and Li (2007)
Treat gynecologic inflammation	*Actinidia eriantha*, *Impatiens balsamina*	Leading role	Chen and Guan (1996)
Treat gastric carcinoma	*Actinidia eriantha*, *Adina rubella*, *Ampelopsis sinica*, *Scutellaria barbata*, *Imperata cylindrica*, *Pteris multifida*, *Scutellaria barbata*	Leading role	Chen and Guan (1996)
Treat breast cancer	*Actinidia eriantha*, *Ampelopsis sinica*, *Dysosma versipellis*, *Arisaema erubescens*	Leading role	Chen and Guan (1996)
Treat leukorrhagia	*Plantago depressa*, *Celosia cristata*, *Phellodendron chinense*, *Actinidia eriantha*, *Lonicera japonica*, *Trema cannabina*, *Lophatherum gracile*, *Juncus effusus*, *Baimuhua*	Supporting role	Lei and Li (2007)
Treat sprain	*Ampelopsis sinica*, *Aralia chinensis*, *Actinidia eriantha*, *Pinus massoniana*, *Tuopihuang*	Supporting role	Lei and Li (2007)
Treat fracture	*Ampelopsis sinica*, *Aralia chinensis*, *Actinidia eriantha*, *Mallotus japonicus*, borneol, indigo naturalis, musk, *Xiyemaotonggen*, *Tuopihuang*	Supporting role	Lei and Li (2007)
Treat furunculosis	*Paulownia tomentosa*, *Actinidia eriantha*	Supporting role	Lei and Li, (2007)
Treat tumor	*Hedyotis diffusa*, *Scutellaria barbata*, *Solanum lyratum*, *Actinidia eriantha*, *Ampelopsis sinica*, *Actinidia chinensis*, *Salvia chinensia*	Supporting role	Lei and Li (2007)
Treat intestinal cancer	*Actinidia eriantha*, *Scutellaria barbata*, *Hedyotis diffusa*, *Cephalotaxus fortunei*, *Rabdosia amethystoides*, *Ampelopsis sinica*, *Huagucao*	Supporting role	Lei and Li (2007)
Treat prolapses of uterus	*Actinidia eriantha*, *Trichosanthes kirilowii*, pork intestine	Supporting role	Lei and Li (2007)
Treat cirrhosis with ascites	*Lygodium japonicum*, *Juncus effusus*, *Adina rubella*, *Gardenia jasminoide*, *Scutellaria barbata*, *Hedyotis diffusa*, *Cynanchum stauntonii*, *Rabdosia amethystoides*, *Artemisia scoparza*, *Kalimeris indica*, *Plantage asiatica*, *Selaginella involens*, *Actinidia eriantha*	Supporting role	Lei and Li (2007)
Treat epigastric pain	*Alpinia japonica*, *Agrimonia pilosa*, *Hypericum japonicum*, *Cyperus rotundus*, *Artemisia argyi*, *Dichondra micrantha*, *Actinidia eriantha*, *Brucea javanica*, *Guomenda*, *Hongqiangmanteng*	Supporting role	Chen and Guan (1996)

## Quality control

For quality control of AE-derived medicines, the Zhejiang Processing Standard of Traditional Chinese Medicine (ZPSTCM) suggested morphological, microscopic and chemical identification (Zhejiang Food and Drug Administration, 2015). According to the requirements of ZPSTCM, moisture shall be not exceed 12% (“Chinese Pharmacopoeia “moisture determination drying method), while the total ash shall be not exceed 7% (“Chinese Pharmacopoeia” ash determination method) (Zhejiang Food and Drug Administration, 2015). What’s more, the bioactive components of AE, including general flavone and total triterpenes, have been identified by ultraviolet spectroscopy. A good linearity of rutin was shown in ranges of 0.00942–0.05650 mg mL^−1^ (*r* = 0.9991). The average recovery rate was 97.33% (RSD = 1.98%). A good linearity of ursolic acid was shown in ranges of 0.004 89–0.01712 mg/ml (*r* = 0.9987) and the average recovery rate was 96.58% (RSD = 1.79%) (Yu et al., 2017). In terms of dried products, the total flavonoids in the form of anhydrous rutin (C_27_H_30_O_16_) and total three pieces in the form of ursolic acid (C_30_H_48_O_3_) should not be less than 1.0% (Yu et al., 2017). What’s more, *via* optimizing the conditions of ultrasonic extraction, the average total flavonoids content in the root of AE could up to 1.058% (Zheng et al., 2012). By optimizing ultrasonic extraction conditions, the total triterpenoid content in the root of AE could reach to 1.19% ± 0.08% (Li et al., 2015). In further study, using the combination of ultrasonic extraction and percolation extraction, the content of triterpenoids in the ethyl acetate extract of the root of AE can be as high as 45% (Zhang et al., 2019). However, adopting only one crude, quantitative marker to ascertain the quality of AE extract may not be convincing enough.

AE root is a traditional medicine used in folk cancer treatment of *she* people, and is widely used in the treatment of gastric cancer, esophageal cancer, breast cancer, nasopharyngeal cancer, and liver cancer, etc. Triterpenoid acid is one of the main active substance (Gong et al., 2016). Guo et al. used HPLC-PDA to compare the contents of 2α,3α,24-trihydroxy-12-ene-28-ursolic acid in the roots of AE from different origins, finding it existed in the roots of AE from producing areas, but the content was different (Li et al., 2015). These results provided reference for the quality evaluation of AE roots from different origins. What’s more, total flavonoids were used as the main index to evaluate the quality of *Actinidia chinensis* Planch, which is indicated that can be used as a candidate index component for evaluation of AE (Shaanxi Food and Drug Administration, 2015).

## Safety

Based on available animal trails, AE administration seems to cause no or little toxicity. According to the results of body weight measurement and microscopic examination of organs (intestinal tract, liver and kidney), there have been no observed toxic effects of AEPS (the total polysaccharide from the root of AE) and its polysaccharides on mice at the maximum dose of 10 mg/kg (Xu et al., 2009a; Xu et al., 2009b). In addition, when the mice were subjected to subcutaneous administration of AEPS at the doses of 0.5–5.0 mg/kg twice a week, no local swelling or hair loss was observed. These results implied that the upper safe dosage of AEPS for humans and animals may be higher than 200 mg/kg (Sun et al., 2009). What’s more, AEPS did not elicit cytotoxicity in RAW264.7 cells at a high concentration of 200 μg/ml; in fact, AEPS at the concentrations of 25–100 μg/ml could even promote the proliferation of RAW264.7 cells (Sun et al., 2015). However, further investigations specifically focusing on the chronic toxicity of AE should be carried out.

## Discussion

*A. eriantha* is a liana species that has been extensively utilized in TCM to treat various diseases in China. Our paper summarizes the existing knowledge on the botanical characteristics, traditional uses, phytochemical properties, pharmacological activities, and toxicity of AE. In classical TCM documents and ZPSTCM, AE is commonly used to treat stomach cancer, colon cancer, cirrhosis with ascites, chronic hepatitis, leukemia, rectal prolapse, hernia and uterine prolapse. Pharmacological studies have demonstrated many bioactivities of AE, including anti-cancer, immunoregulatory, anti-inflammatory, anti-angiogenic, neuroprotective and antioxidant activities. However, many traditional uses of AE have not been supported by pharmacological research. To date, over 104 chemical components have been extracted from AE, among which triterpenoids and polysaccharides are the main bioactive substances.

Our current understanding of the phytochemical properties and pharmacological activities of AE is insufficient. Firstly, although AE administration exhibited almost no toxicity in most animal studies, its long-term toxicity should be further assessed. The possible adverse effects and biotoxicity of AE extracts and their active components should be evaluated when they are used for *in vitro*, *in vivo*, or clinical studies. Secondly, most pharmacological investigations on AE have been performed on its crude extracts and fractions, of which few have been analyzed for phytochemical properties. For example, AE polysaccharides possess antitumor, immunomodulatory, and anti-inflammatory activities, but how these activities of AE polysaccharides correlate with their structural characteristics remains clear. Thus, these bioactive components of AE should be isolated and studied for their molecular mechanisms, bioavailability, and pharmacokinetic characteristics in future research. Thirdly, although AE-derived polysaccharides have been shown to possess similar anti-tumor activity as ethyl acetate fraction (representative compounds: triterpenoids), the mechanisms underlying absorption, distribution, metabolism, and excretion, as well as the synergistic or antagonistic effects between the two constituents are unknown and should be further studied. Moreover, the synergistic anti-tumor and therapeutic action, as well as other pharmacological activities, such as anti-diabetic, renal protective, and neuro-protective effects, should be taken into consideration in future study. Last but not the least, the idea that AE is responsible for cirrhosis with ascites, chronic hepatitis, rectal prolapse, hernia and uterine prolapse should be validated by modern pharmacological research; once validated, the mechanisms underlying these bioactivities of AE should be further explored. Given that most of the existing research was conducted at the cellular level, more *in vivo* studies adopting animal models and clinical samples are needed to testify the efficacy of AE in the treatment of eat stomach cancer, colon cancer, cirrhosis with ascites, chronic hepatitis, leukemia, rectal prolapse, hernia and uterine prolapse. Nevertheless, a lot of the traditional uses of AE have not been the focus of recent research, which warrant further investigations.

## Glossary

ABTS: 2, 2′-azino-bis(3-ethylbenzothiazoline-6-sulfonic acid)
AE: *Actinidia eriantha*
Bax: BCL2-associated X
Bcl-2: B-cell lymphoma-2
CAM: Chick embryo chorioallantoic membrane
CAT: Catalase
CD3: Cluster of differentiation 3
ConA: Concanavalin A
CXCL: Chemokines
DPPH: 1,1-diphenyl-2-picrylhydrazyl
EA-EER: Ethyl acetate fraction of AE root
FRAP: High ferric reducing ability of plasma
GSH/GSSG: L-Glutathione
HPLC: High purity liquid chromatography
HPLC-PDA: High purity liquid chromatography-photo-diode array
IL: Interleukin
INF: Interferon
lncRNA: Long noncoding RNA
LPS: Lipopolysaccharide
mAb: Monoclonal antibody
miRNA: Micro RNA
mRNA: MessengerRNA
n-BuOH: Normal butanol
NK cell: Natural killer
ORAC: Oxygen-radical absorbance capacity
OVA: Ovalbumin
PCDH10: Protocadherin 10
PD-L1: Programmed death 1 ligand 1
PD-L2: Programmed death 1 ligand 2
RSD: Rlative sandard deviation
SOD: Superoxide dismutase
TCM: Traditional chinese medicine
TNF-α: Tumor necrosis factor α
VEGF-A: Vascular endothelial growth factor A
VEGFR-2: Vascular endothelial growth factor receptor 2
ZPSTCM: Zhejiang processing standard of traditional chinese medicine
